# CD4^+^T cell specific B7-H1 selectively inhibits proliferation of naïve T cells and Th17 differentiation in experimental autoimmune encephalomyelitis

**DOI:** 10.18632/oncotarget.21357

**Published:** 2017-09-28

**Authors:** Sheng-Jia Shi, Mei-Ling Ding, Li-Juan Wang, Jie-Heng Wu, Dong-Hui Han, Guo-Xu Zheng, Zhang-Yan Guo, Wen-Jin Xi, Wei-Jun Qin, An-Gang Yang, Wei-Hong Wen

**Affiliations:** ^1^ State Key Laboratory of Cancer Biology, Department of Immunology, Fourth Military Medical University, Xi’an, 710069, Shaanxi Province, P.R. China; ^2^ Department of Urology, Xijing Hospital, Fourth Military Medical University, Xi’an, 710032, Shaanxi Province, P.R. China; ^3^ Reproduction Medicine Center, No. 202 Hospital of PLA, Shenyang, 11000, Liaoning Province, P.R. China; ^4^ State Key Laboratory of Cancer Biology, National Clinical Research Center for Digestive Disease, Xijing Hospital of Digestive Diseases, Fourth Military Medical University, Xi’an, 710032, Shaanxi Province, P.R. China; ^5^ Department of Dermatology, First Affiliated Hospital of Xi’an Jiaotong University, Xi’an 710061, Shaanxi Province, P.R. China

**Keywords:** B7-H1, EAE, Th17 cells, multiple sclerosis

## Abstract

It is widely acknowledged that interleukin 17-producing T helper (Th17) cells are critically participant in the pathogenesis of multiple sclerosis. In the current study, we identified that the expression of CD4^+^T cells specific co-inhibitory molecule B7-homologue 1(B7-H1) in spleenocytes and mononuclear cells isolated from brains and spinal cord were positive correlated with Th1 and Th17 cells generation and disease severity in experimental autoimmune encephalomyelitis (EAE). Furthermore, B7-H1 transgenic mice developed milder EAE symptoms and fewer Th17 cells than B7-H1 wild type mice. We also found the proliferation of naïve CD4^+^CD62^+^T cells isolated from B7-H1 transgenic mice was inhibited. And naïve T cells isolated from B7-H1 transgenic mice produced fewer Th17 cells than WT mice in Th17-polarizing conditions, but the Th1, Th2, and inducible Treg differentiation were the similar in naïve T cells isolated from B7-H1 transgenic mice and WT mice. In conclusion, our study show CD4^+^T cells specific B7-H1 is a slective inhibitor in proliferation of naïve T cells, Th17 differentiation and pathogenesis of multiple sclerosis.

## INTRODUCTION

Multiple sclerosis (MS) is a organ-specific autoimmune disease, which characterized by chronic inflammatory demylination of certral nervous system (CNS) [1]. But, due to lack of sensitive biomarker and limited understanding of its pathogenesis, it is difficult to effectively diagnose or treat MS [2]. CD4+ T-cell mediated autoimmunity against a putative myelin autoantigen has long been recognized as a essential aspects of MS pathogenesis [3]. The number of interleukin 17-producing T helper (Th17) cells was proved to be pivotal and made a great contribution to protection against microbial pathogens in the development of MS and the animal model of MS, experimental autoimmune encephalomyelitis (EAE) [4, 5]. Recent evidence indicates that suppression of Th17 differentiation *in vivo* could ameliorate EAE [6, 7]. More imorptantly, Th17 cells have been found significantly upregulated in lesions of CNS from ptients with MS [8]. However, the understanding concerning underlying mechanisms of T-cell polarization into Th 17 subtypes in the development of MS is still at its early stage.

B7 homologue 1 (B7-H1) also known as programmed death ligand-1 (PD-L1) is a member of the B7 family. B7-H1 could suppresses T-cell immune activity and restricts tumor cell killing by binding to its receptor PD-1 [9]. B7-H1 expression on tumor cells was proved to significantly correlate with poor prognosis in multiple types of cancers [10–12]. Thus, B7-H1 were frequently use as a target in immune checkpoint blockade [13, 14]. In addition, the engagement of B7-H1 with PD-1 could suppress the proliferation of autoreactive T cell and inhibit secretion of inflammatrory cytokine in EAE [15]. Howerver, the therapeutic potential of B7-H1 for MS and the precise mechamism are still largely unknown.

In the current study, we report that the CD4^+^T cells specific B7-H1 is critical in regulating Th17 differentiation and contribute to the pathogenesis of MS. Our results provide evidence that there is a significant positive correlations among CD4^+^T cells specific B7-H1 and Th17 production and EAE development. Furthermore, we also found CD4^+^T cells specific B7-H1 could selectively inhibit naïve T cell proliferation and Th17 differentiation during EAE development. Collectively, our study indicates that CD4^+^T cells specific B7-H1 may be a promising targets for control of Th17 differentiation in MS and EAE.

## RESULTS

### Expression of Th1 and Th17 cells during EAE development

**Figure 1:**
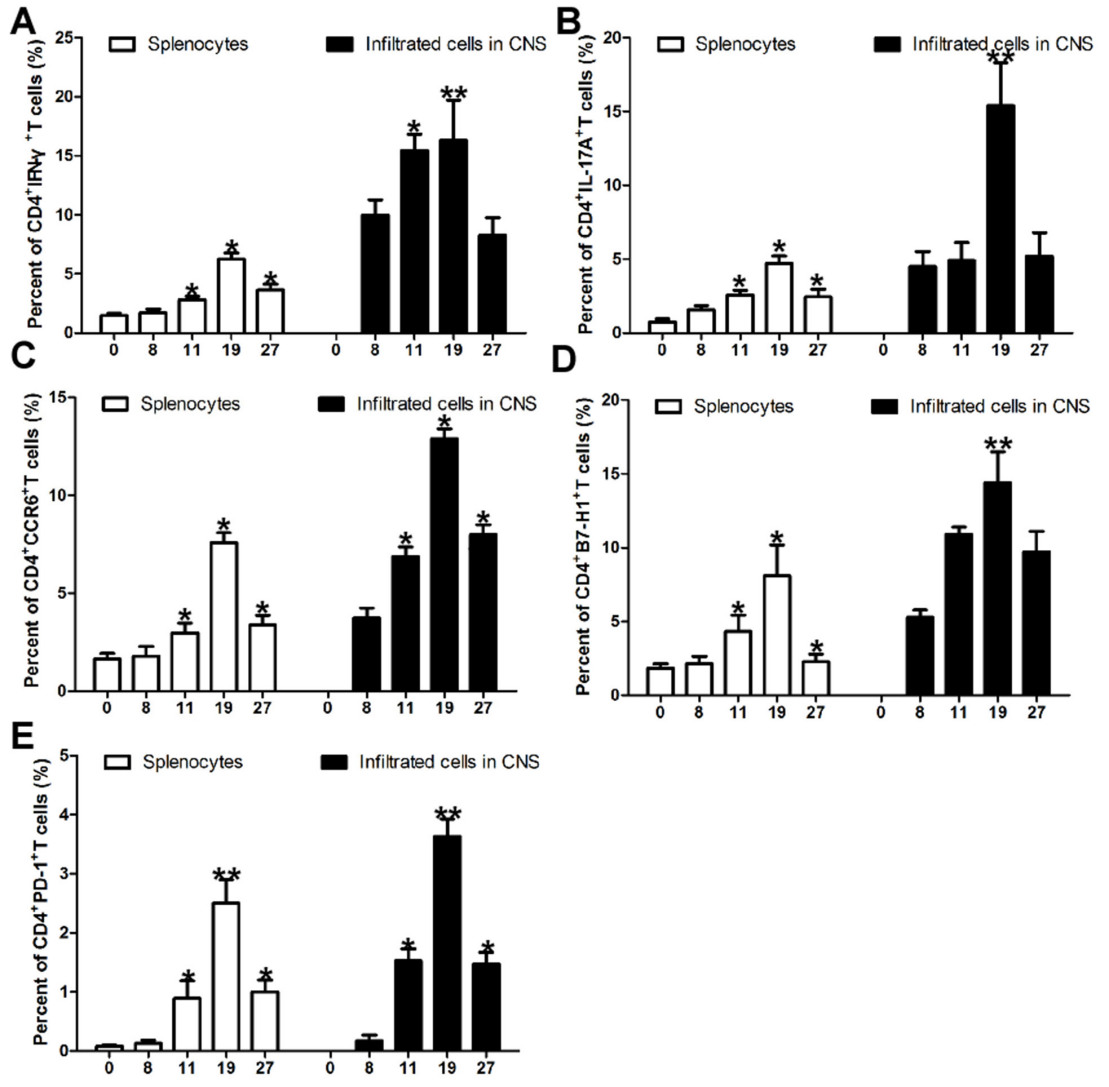
Expression of Th1 cells, Th17 cells, PD-1 and B7-H1 positive CD4^+^T cells during EAE development **(A)** Intracellular staining of IFN-γ in the splenocytes and mononuclear cells infiltrated in CNS during EAE development. Intracellular staining of IFN-γ in the spleenocytes and mononuclear cells infiltrated in CNS indicate percent cells in the CD4^+^ gate. **(B)** Intracellular staining of IL-17A in the splenocytes and mononuclear cells infiltrated in CNS during EAE development. Intracellular staining of IL-17A in the spleenocytes and mononuclear cells infiltrated in CNS indicate percent cells in the CD4^+^ gate. **(C)** Expression of CD4^+^CCR6^+^ cells in the splenocytes and mononuclear cells infiltrated in CNS during EAE development. **(D)** Expression of CD4^+^T cell specific B7-H1 in the splenocytes and mononuclear cells infiltrated in CNS during EAE development. **(E)** Expression of CD4^+^T cell specific PD-1 in the splenocytes and mononuclear cells infiltrated in CNS during EAE development. Five female B7-H1 WT mice 6-8 weeks of age were used to established EAE model. ^*^P < 0.05 and ^**^P < 0.01. (Student's t-test). Data are from three independent experiments (mean and s.e.m).

In order to investigate the expression of Th1 and Th17 cells in EAE development, we detect IFN-γ and IL-17A expression in CD4^+^T cells during the progression of EAE. As the EAE clinical score increasing from day 0 to day 19 after immunization of encephalitogenic peptide of myelin oligodendrocyte glycoprotein consisting of amino acids 35-55 (MOG (35-55)), CD4^+^IFN-γ^+^ (Figure 1A and Supplementary Figure 1A) and CD4^+^IL-17A^+^ (Figure 1B and Supplementary Figure 1A) cells in splenocytes and mononuclear cells infiltrated in central nervous system (CNS) were also increasing. But when the EAE sypmtoms were remiting since day 19 after immunization of MOG (35-55), IFN-γ (Figure 1A and Supplementary Figure 1A) and IL-17A (Figure 1B and Supplementary Figure 1A) specific CD4^+^ T cells isolated from spleen or CNS were also decreasing. Specifically, CD4^+^CCR6^+^ cells in splenocytes and mononuclear cells isolated from brains and spinal were also positively associated with EAE scores during EAE development (Figure 1C and Supplementary Figure 1B).

### Expression of B7-H1 and PD-1 on CD4+T cells during EAE development

**Figure 2:**
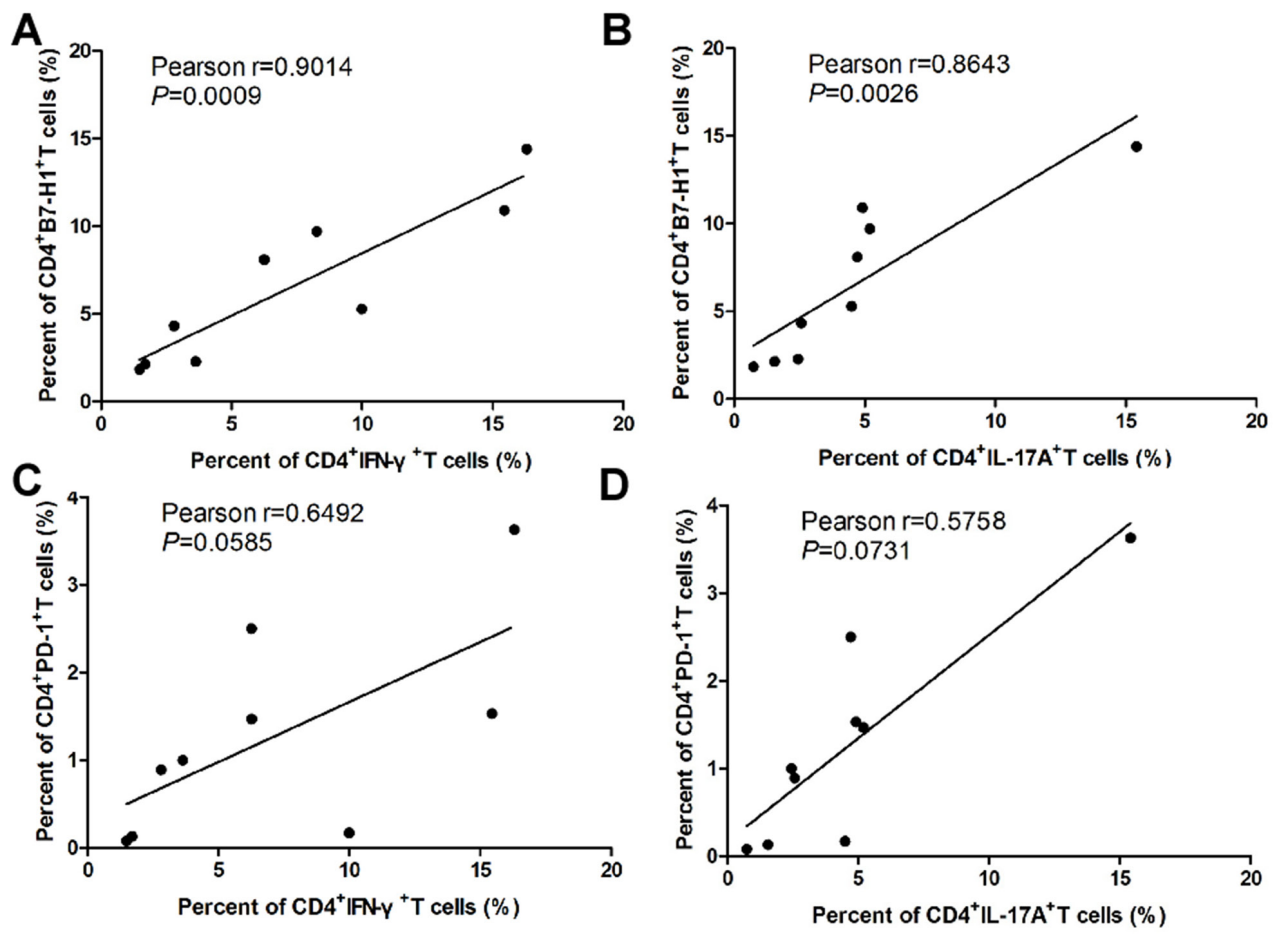
Relationship between of B7-H1 and PD-1 on CD4+T cell and Th1 and Th17 cells during EAE development **(A)** Relationships between of B7-H1^+^CD4^+^T cells and IFN-γ^+^CD4^+^T cells in splenocytes and mononuclear cells infiltrated in CNS during EAE development. **(B)** Relationships between of B7-H1^+^CD4^+^T cells and IL-17A^+^CD4^+^T cells in splenocytes and mononuclear cells infiltrated in CNS during EAE development. **(C)** Relationships between of PD-1^+^CD4^+^T cells and IFN-γ^+^CD4^+^T cells in splenocytes and mononuclear cells infiltrated in CNS during EAE development. **(D)** Relationships between of PD-1^+^CD4^+^T cells and IL-17A^+^CD4^+^T cells in splenocytes and mononuclear cells infiltrated in CNS during EAE development. Five female B7-H1 WT mice 6-8 weeks of age were used to established EAE model. ^*^P < 0.05 and ^**^P < 0.01. (Pearson correlation analysis).

In order to further investigate the expressions of B7-H1 and its receptor PD-1 in EAE development, we detected CD4^+^T cells specific B7-H1 and PD-1 during the progression of EAE by flow cytometry. As the EAE clinical scores increasing from day 0 to day 19 after immunization of MOG (35-55), the expression of CD4^+^T cells specific B7-H1 (Figure 1D and Supplementary Figure 2A and 2C) and PD-1 (Figure 1E and Supplementary Figure 2B and 2D) isolated from spleen and CNS were also increasing. But when the EAE sypmtoms were remiting since day 19 after immunization of MOG (35-55), the expression of CD4^+^T cells specific B7-H1 (Figure 1D and Supplementary Figure 2A and 2C) and PD-1 (Figure 1E and Supplementary Figure 2B and 2D) isolated from spleen and CNS were also decreasing. More importantly, pearson correlation analysis showed the expression of CD4^+^T cells specific B7-H1 was positively associated with Th1 (γ=0.9014, P=0.0009; Figure 2A) and Th17 (γ=0.8643, P=0.0026; Figure 2B) cells during EAE development. But CD4^+^T cells specific PD-1 was not positively associated with Th1 (γ=0.6492, P=0.0585; Figure 2C) and Th17 (γ=0.5758, P=0.0731; Figure 2D) cells during EAE development.

### Regulation of EAE development by B7-H1

**Figure 3:**
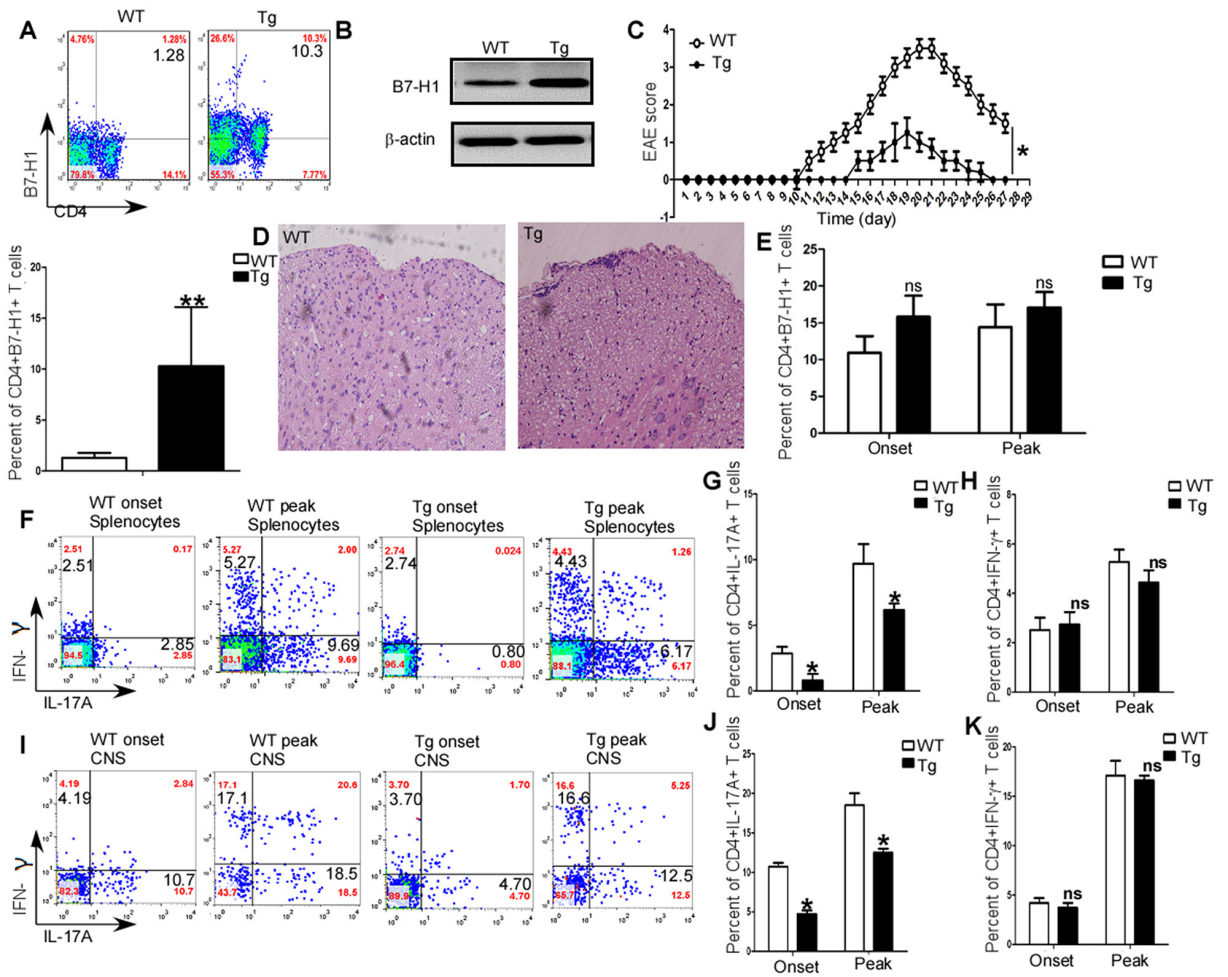
Regulation of EAE and generation of Th1/Th17 *in vivo* by B7-H1 **(A)** RT-PCR analysis of B7-H1expression in splenocytes of B7-H1 transgenic mice. **(B)** Flow cytometry analysis of CD4^+^T cell specific B7-H1expression in splenocytes of B7-H1 transgenic mice and WT mice. **(C)** Clinical scores for EAE in B7-H1 transgenic mice and WT mice. **(D)** Histology of paraffin section of spinal cords isolated from B7-H1 transgenic mice and B7-H1 WT mice on day19 after immunization. **(E)** Intracellular staining of IL-17A and IFN-γ in the splenocytes and mononuclear cells infiltrated in CNS on day 11 (onset) and day 19 (peak). Numbers in quadrants indicate percent cells in the CD4+ gate. Five female B7-H1 WT and trangenic mice 6-8 weeks of age were used to established EAE model, respectively.^*^P < 0.05 and ^**^P < 0.01 (Student's t-test for parametric data and Mann–Whitney test for nonparametric data). Data are from three experiments (mean and s.e.m)

In order to further investigate the funcions of B7-H1 in the development of EAE, we detected the differences in symptoms and pathologies between B7-H1 wild type (WT) and B7-H1 transgenic C57/B6 mice. As shown in Figure 3A (RT-PCR and gel electrophoresis), B7-H1 expression was significanlty upregulated in B7-H1 transgenic mice. Furthermore, CD4^+^T cell specific B7-H1 was also upregulated in splenocytes of B7-H1 transgenic mice (*P*<0.01; Figure 3B). After immunization of MOG (35-55), B7-H1 WT mice developed severe EAE, whereas B7-H1 transgenic mice had somewhat mild EAE (*P*<0.05, Figure 3C). Histological analysis of spinal cord sections also showed B7-H1 transgenic mice developed mild inflammatory infiltration (Figure 3D). Though CD4+T cell specific B7-H1 expressions were similar in mononuclear cell infiltrated in CNS of B7-H1 transgenic mice and WT mice (*P*>0.05; Figure 3E), Th17 cells in splenocytes (*P*<0.05; Figure 3F & 3G) and mononuclear cell infiltrated in CNS (*P*<0.05; Figure 3I & 3J) were decreased in B7-H1 transgenic mice. However, Th1 cells in splenocytes (*P*>0.05; Figure 3F & 3H) and mononuclear cell isolated from brains and spinal cords (*P*>0.05; Figure 3I & 3K) were similar in B7-H1 transgenic mice and WT mice.

### Inhibition of Th17 differentiation by B7-H1

**Figure 4:**
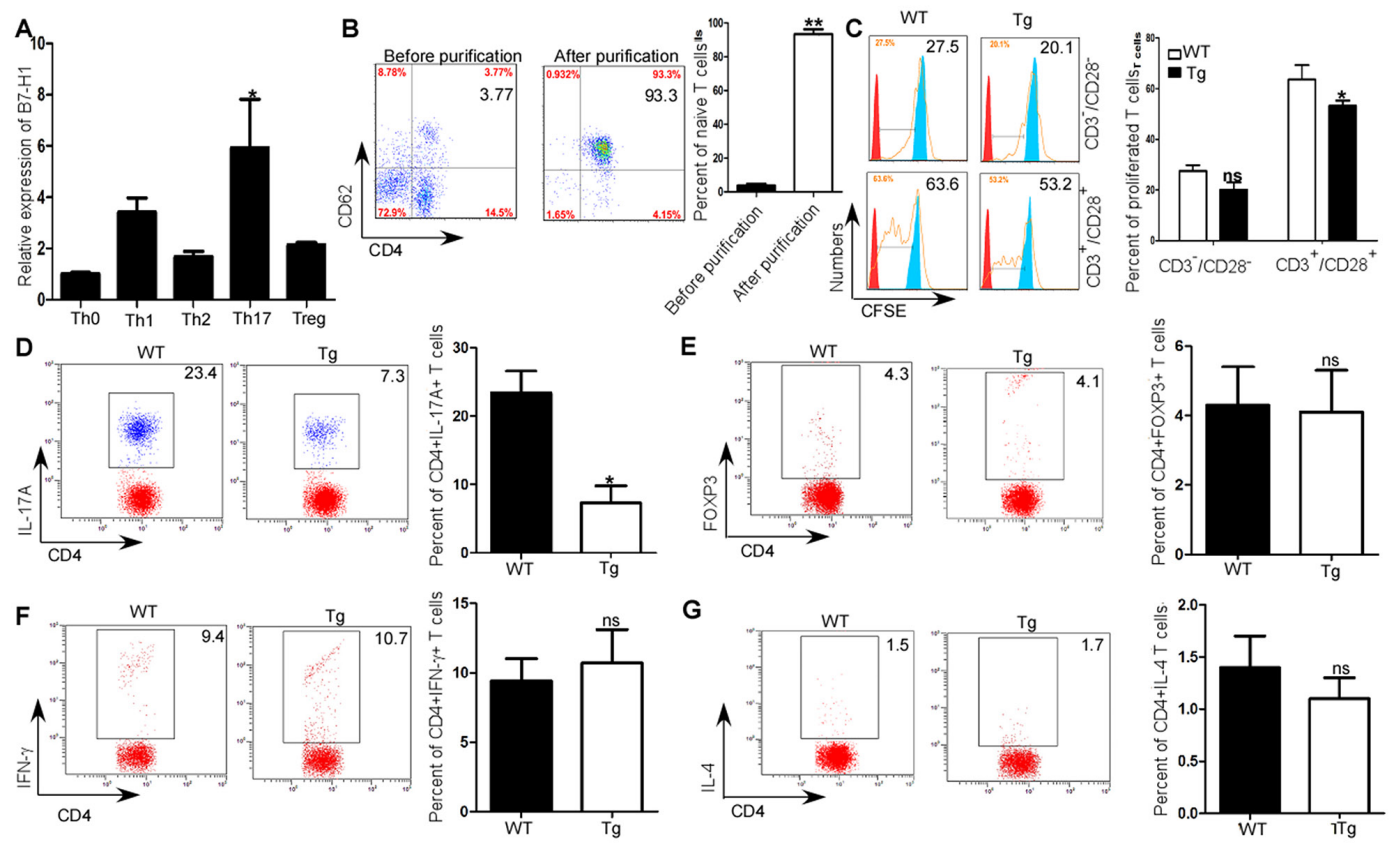
Promotion of *in vitro* Th17 differentiation by B7-H1 **(A)** qRT-PCR analysis of B7-H1 expression in Th0, Th1, Th2, Th17, and inducible Treg cells. **(B)** Flow cytometry analysis of expression of CD4^+^CD62^+^ T cells isolated from spleens before and after naïve T cell purification. **(C)** CFSE analysis of proliferation of naïve CD4^+^ CD62^+^ T cells obtained from B7-H1 transgenic and WT mice and activated by anti-CD3 and anti-CD28. **(D)** Intracellular staining of IL-17A of naïve CD4^+^ CD62^+^ T cells obtained from B7-H1 WT and transgenic mice and culture for 4d in Th17 polarizing conditions. Numbers in outlined areas (D) indicate percent IL-17+ cells in the CD4+ gate. **(E)** Intracellular staining of Foxp3 of naïve CD4^+^ CD62^+^ T cells obtained from B7-H1 WT and transgenic mice and culture for 4d in inducible Treg polarizing conditions. Numbers in outlined areas (E) indicate percent Foxp3+ cells in the CD4+ gate. **(F)** Intracellular staining of Foxp3 of naïve CD4^+^ CD62^+^ T cells obtained from B7-H1 WT and transgenic mice and culture for 4d in inducible Th1 polarizing conditions. Numbers in outlined areas (F) indicate percent IFN-γ+ cells in the CD4+ gate. **(G)** Intracellular staining of Foxp3 of naïve CD4^+^ CD62^+^ T cells obtained from B7-H1 WT and transgenic mice and culture for 4d in inducible Th2 polarizing conditions. Numbers in outlined areas (G) indicate percent IL-4^+^ cells in the CD4^+^ gate. *P* < 0.05 and ^**^P < 0.01 (Student's t-test). Data are from three experiments (mean and s.e.m).

As both CD4+T cell specific B7-H1 and Th1/Th17 were positive correlated with EAE scores, and CD4^+^T cell specific B7-H1 was positively correlated with Th1 and Th17 expression during EAE development. Thus, we further investigated the roles of CD4^+^T cell specific B7-H1 in Th cells differentiation. By comparing B7-H1 expression in all subsets of helper T cells with that in naïve CD4^+^T cells, we found large amounts of B7-H1 preferentially in Th17 cells but relatively small amounts in Th1, Th2 and inducible Treg cells (*P*<0.05; Figure 4A). In order to further investigate the effects of CD4^+^ T cell specific B7-H1 on the proliferation and Th differentiation, we sorted CD4^+^CD62^+^ T cells from spleens of B7-H1 WT and transgenic mice (*P*<0.05; Figure 4B) to get high purity naïve T cells. Compared with native T cell isolated from wild type mice, we found the proliferation of native T cell isolated from B7-H1 transgenic mice was inhibited after stimulating with CD3/CD28 antibody (*P*<0.05; Figure 4C). We then primed the cells for Th17 differentiation in *vitro*, and found more Th17 cells were produced by naïve CD4^+^T cells from B7-H1 WT mice and fewer were produced by those from B7-H1 transgenic mice (*P*<0.05; Figure 4D). But, Th1(*P*>0.05; Figure 4E), Th2 (*P*>0.05; Figure 4F), inducible Treg (*P*>0.05; Figure 4G) cells pruduced by naïve CD4+T cells purified from B7-H1 trasngenic mice was the similar as WT mice.

## DISCUSSION

Using EAE model of B7-H1 transgenic mice and B7-H1 WT mice, our study provided evidence that CD4+T cell specific B7-H1 could significantly inhibit proliferation of naïve T cells, Th17 lineage differentiation and help to control T-cell mediated CNS auto immunity. We found both CD4^+^T cells specific B7-H1 and its receptor PD-1 were upregulated during EAE development. And CD4^+^T cells specific B7-H1 was positive correlated with Th1 and Th17 production. In additon, B7-H1 transgenic mice exhibited an delayed disease onset, significantly modest EAE severity, and fewer Th17 cells production. More importantly, compared with WT mice, the proliferation of naïve CD4^+^CD62^+^ helper T cells isolated from spleen of B7-H1 transgenic mice was inhibited. Furthermore, naïve T cells purified from B7-H1 transgenic mice produced fewer Th17 cells than B7-H1 WT mice in Th17-polarizing conditions.

It had been provided substantial evidence that Th17 cells had significant role in MS. Up-regulation of Th17 cells and IL-17A was observerd in the CNS of MS patients, especially in chronic active lesions and acute lesions [16, 17]. More importantly, inhibition of Th17 cell differentiation could significantly ameliorated MOG (35-55)-induced EAE [18, 19]. For example, CD226 pAb administration was found to reduced onset of EAE by inhibiting Th1/Th17 production [20]. Furthermore, huma truncate IL12rβ1-Fc fusion protein could ameliorate EAE and suppress demyelination in CNS by reducing production of Th1 and Th17-polarized proinflammatory cytokines [21]. And betulinic acid derivative, SH479, was found to have inhibitory effect on Th17 differentation, thus could ameliorate clinical and histological signs of EAE. In our study, we found CD4^+^T cells specific B7-H1 could significantly inhibit Th17 differentiation *in vivo* and *in vitro*. In addition, compare with B7-H1 WT mice, B7-H1 trasgenic mice developed mild EAE symptoms and had fewer Th17 cells infiltrated in CNS and spinal cord. Our study provides new evidence to the relationships between Th17 cell differentiation and EAE onset and development. Furthermore, we also found the expression of CD4^+^T cells specific B7-H1 was also positively correlated with Th1 production *in vivo* during EAE development, but CD4^+^T cells specific B7-H1 had little effects on Th1 differentiation *in vitro*. And, the prudction of Th1 cells in B7-H1 transgenic mice was the almost the same as WT mice. The different signaling pathway to regulate Th1 and Th17 differentiation should account for this discrepancy, more specific underlying mechanism needed further investigated.

B7-H1 had been proven to be a key regulator of maintenance of immune tolerance, and the function of B7-H1 on atigen-presenting cells (APCs) in MS and EAE has been well established [22]. B7-H1 konckout mice displayed an significantly exacerbated EAE severity and accelerated disease onset [23]. B7-H1 was also found significantly up-regulated in lesions of MS, which colocalized with microglia/macrophage cell markers or astrocyte [23, 24]. Moreover, glial cells which expressed sufficient and functional B7-H1 could inhibit CD8+T cell response effectively [24]. B7-H1 expression on B cells and monocytes were significantly augmented in stable MS patients, and B7-H1 expression on immune cells was reduced in treated MS patients [25]. However, the association of CD4^+^T cells specific B7-H1 and MS development was not fully investigated. A recent published study indicate that B7-H1 ablation on MOG-specific CD4^+^T cell significantly amplified proinflammatory and lytic effector functions, such as IFN-γand granzyme A and B production, but Th 17 response remained unchanged between B7-H1 KO T cells and B7-H1 WT T cells [26]. In contrast these findings, we found upregulation of CD4^+^T cells specific B7-H1 could significantly selectively inhibit Th17 differentiation during EAE development but had little effects on Th1 differentiation, which was in line with a study which argued B7-H1 fusion protein could slectively controlled Th17 cells differentiation, but not Th1 and Th2 [27]. The discrepancy among our study and previous studies maybe due to the difference of animal model or research strategy.

The funtion of CD4^+^T cells specific B7-H1 on Th cell differentiation was still in dispute. It had been found that B7-H1 expressed by CNS myeloid APCs more selectively suppresses Th1 differentiation than Th17 differentiation in EAE [28]. In addition, B7-H1 also down-regulated acute graft-vs-host disease through selective modulation of IFN-γ production [29]. And bolokade of B7-H1 on dendritic cells could result in enhanced T cell proliferation and IFN-γproduction [30]. Blocking B7-H1 expression on human endothelial cells with morpholino antisense olignucleotides could augment IFN-γ expression. All these data indicated B7-H1 may primarily affects production of IFN-γ or Th1 differentiation. But a recent study found B7-H1 on CD4+T cells had no effect on Th1 differentiation and did not generally interfere with effector T cell generation [27]. However, it was also found that B7-H1 expressed on dentrict cells could control generation and function of inducible Treg cell development in EAE [31]. In our study, we found large amounts of CD4^+^T cells specific B7-H1 preferentially in Th17 cells but relatively small amounts in Th1, Th2 and inducible Treg cells. And we provided evidence of negative effects of CD4^+^T cells specific B7-H1 on Th17 diffrentiation by performing naïve T cell differentiation assays. More importantly, we found CD4^+^T cells specific B7-H1 could inhibit Th17 cell differentiation, which indicate B7-H1 maybe function as a receptor rather than merely a ligand for PD-1. Typically, B7-H1 expressed on APCs functions as a ligand of PD-1 to pass inhibitory signals, dwon or terminate T cell response [32]. But, in our study, we found CD4^+^T cells specific B7-H1 could selectively inhibit proliferation of naïve T cells and Th17 differentiation, which indicates B7-H1 maybe function as a receptor in the regulation of Th17 differentiation. Our study deepens our understanding concerning the function and effects of B7-H1 on Th cell differentiation. Though, many studies also indicate B7-H1 may not only a ligand for PD-1 but also may function as a receptor, the downstream effector genes and signalling pathway of B7-H1 are still largely unkown. Further investigations are needed to clarify the underlying mechamis of B7-H1 functions as a receptor and inhibits Th cell differentiation.

In conclusion, we demonstrated that the expression of B7-H1 on CD4+T cells was up-regulated as the EAE clinical scores increased and down-regulated when EAE symptoms relieved, which indicated B7-H1 palys an important role in mediating clinical symptomos and maintaing the pathological process of EAE. In addtion, we found B7-H1 transgenic mice developed a modest EAE symptoms and had fewer Th17 cells infiltrated in CNS than B7-H1 WT mice. More importantly, we revealed B7-H1 could selectively inhibit proliferation and Th17 differentiation of CD4^+^T cells *in vitro*. Our results indicate B7-H1 could be pivital inhibitor of T-cell mediated autoimmunity in MS and a promosing therapeutic target for MS.

## MATERILAS AND METHODS

### Mice

B7-H1 trangenic mice in C57/B6 background were established and kindly offerd by Professor Wang Jian from department of neurobiology, Fourth Military Medical University [33]. B7-H1 WT mice in C57/B6 mice of 7 weeks were purchase from Shanghai Laboratory Animal research center (Chinese Acadamy of Sciences). Protocols for animal experiments were approvel by institutional animal use committee of Fourth Military Medical University. All the mice were maintained under specific pathogen free condition at experimental animal center of Fourth Military Medical University.

### EAE induction and evalution

The encephalitogenic peptide MOG (35-55)(MEVGWYRSPFSRVVHLYRNGK) (GLBiochem) used to induce EAE had a purity of 95%. Female B7-H1 WT or transgenic mice 6-8 weeks of age were injected subcutaneously with 200μg MOG (35-55) and complete freund's adjuvant (CFA) containing 200μg of H37RA (BD Bioscience) in the posterior right and left flank. Pertussis toxin (Calbiochem) in PBS was injected intraperitoneally on day 0 and day 2. The mice were assigned a scores of EAE on a scale of 0-5 as follows [34]: 0. normal; 1. Paralyzed tail; 2. Moderate hind-limb weakness or mild ataxia;3. Severe hind-limb weakness;4.paraplegia with weakness or paralysis;5. Moribund state or death.

For analysis of CNS infiltrates, brain and spinal cord tissues were colletected from perfused mice and mononuclear cells were prepared by Percoll gradient centrifugation. For histological analysis, brain and spinal cord tissues were immediatedly fixed in 4% paraformaldehyde.

### T cell purification and Th17 differentiation assays

Naïve CD4^+^CD62L^+^ helper T cells were purified by magnetic cell sorting from spleens (CD4+T cell isoliation kit, mouse, Miltenyi Biotec, Auburn, CA). Sorted cells were stimulated with anti-CD3(5μg/mL, soluble, 145-2C11, BD Biosciences) and anti-CD28 (2μg/mL, soluble, 37.51, BD Biosciences) and were induced to differentiation into Th17 cells by supplementation with TGF-β1(5ng/mL, Peprotech), IL-1β (30ng/mL, Peprotech), IL-6 (30ng/mL, Peprotech), tumor necrosis factor (10ng/mL, Peprotech), anti-IL-4 (XMG1.2, BD Bioscience) and anti-IL-IFN-γ (11B11, BD Bioscience).

### T cell proliferation assays

Naïve CD4^+^CD62L^+^ cells were stimulated with anti-CD3(5μg/mL, soluble, 145-2C11, BD Biosciences) and anti-CD28 (2μg/mL, soluble, 37.51, BD Biosciences), and then re-suspended to 1×106 cell/mL in PBS with 10% bovine serum albumin. Cells was added 5μM CFSE solution (Abnova) and incubate 10min at 37°C;. Then, cells was added 5 mL 40% cold bovine serum albumin and incubation on ice for 10min. Cells were then washed twice and resuspended in RPMI 1640 with 10% bovine serum albumin. T cell proliferation was analyzed by flow cytometry after 96h.

### Intracellular staining and flow cytometry

Cells obtained from splenocytes or CNS infiltrateds of mice with EAE were restimulated with PMA (50ng/mL, Sigma), ionomycin (750ng/mL, Sigma) and brefeldin A (BD Biosciences). Cells were then stained with FITC-anti-CD4 at 4°C for 30min. After surface staining, cells were resuspended in Fixation/permeabilization sulution (Cytofix/cytoperm kit; BD Bioscience) and incubated at romm tempeature for 20min. Then cells were staned with PE-anti-IL-17A and APC-anti-IFN-γ at 4°C for 30min. FACS Calibur was used for flow cytometry analysis.

### Statistics

A two-tailed Student's t-test was applied for statistical comparison of two groups and a Mann–Whitney test for nonparametric data (EAE scoring). Pearson correlation analysis was applied for the statistical correlation of two groups. A *P* value of 0.05 or less was considered significant.

## SUPPLEMENTARY MATERIALS FIGURES



## Abbreviations

MS: Multiple sclerosis
CNS: Certral nervous system
Th17: T helper type 17
EAE: Experimental autoimmune encephalomyelitis
B7-H1: B7 homologue 1
PD-L1: Programmed death ligand-1
MOG (35-55): Myelin oligodendrocyte glycoprotein consisting of amino acids 35-55
