# Supplementary material for: CD4+T cell specific B7-H1 selectively inhibits proliferation of naïve T cells and Th17 differentiation in experimental autoimmune encephalomyelitis
Source: Oncotarget. 2017 Sep 28;8(52):90028–36. doi: 10.18632/oncotarget.21357 (PMC5685729; doi:10.18632/oncotarget.21357)
Supplement: Supplementary file 1 [file oncotarget-08-90028-s001.pdf]

CD4⁺T cell specific B7-H1 selectively inhibits proliferation of naïve T cells and Th17 differentiation in experimental autoimmune encephalomyelitis

SUPPLEMENTARY MATERIALS

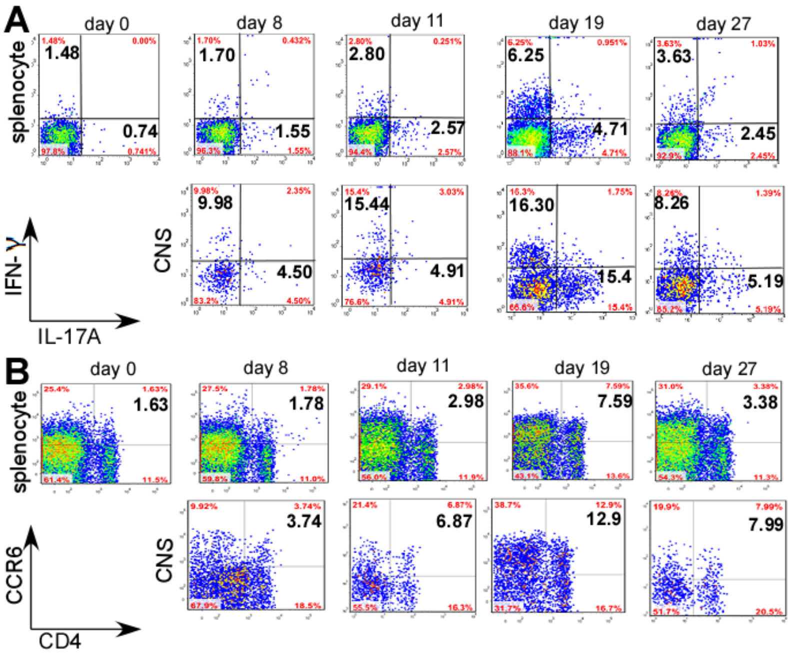

Supplementary Figure 1: Expression of Th1 cells and Th17 cells during EAE development. (A) Intracellular staining of IL-17A and IFN-γ in the splenocytes and mononuclear cells infiltrated in CNS during EAE development. Intracellular staining of IL-17 and IFN-γ in the spleenocytes and mononuclear cells infiltrated in CNS indicate percent cells in the $CD4^+$ gate. **(B)** Expression of $CD4^+CCR6^+$ cells in the splenocytes and mononuclear cells infiltrated in CNS during EAE development. EAE score: day0:0, day8:0, day 11: 0.83±0.16, day19: 3.08±0.45, day27:1.51±0.43. Five female B7-H1 WT mice 6-8 weeks of age were used to established EAE model. $^*P < 0.05$ and $^{**}P < 0.01$. (Student's t-test). Data are from three independent experiments (mean and s.e.m).

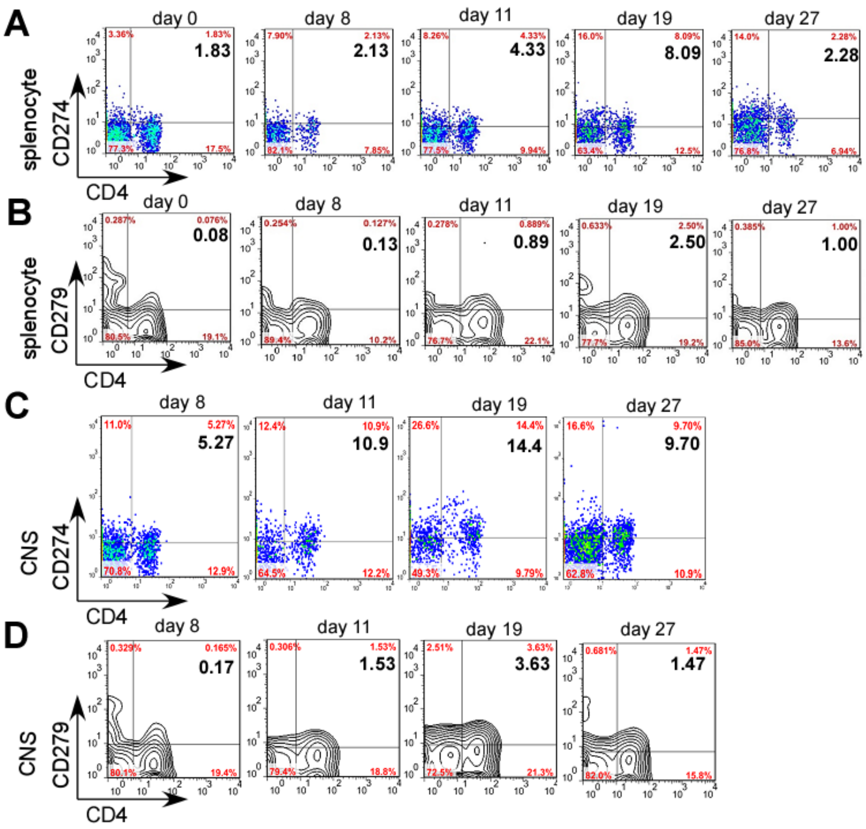

Supplementary Figure 2: Expression of CD4$^+$ specific PD-1 and B7-H1 positive CD4$^+$T cells during EAE development. **(A)** Expression of B7-H1 on CD4$^+$T cells in the splenocytes during EAE development. **(B)** Expression of PD-1 on CD4$^+$T cells in the splenocytes during EAE development. **(C)** Expression of B7-H1 on CD4$^+$T cells in mononuclear cells infiltrated in CNS during EAE development. **(D)** Expression of PD-1 on CD4$^+$T cells in mononuclear cells infiltrated in CNS during EAE development. Five female B7-H1 WT mice 6-8 weeks of age were used to established EAE model.$^*P < 0.05$ and $^{**}P < 0.01$. (Student's t-test). Data are from three independent experiments (mean and s.e.m).
